# GluN2B-Containg NMDA Receptors on Adult-Born Granule Cells Contribute to the Antidepressant Action of Fluoxetine

**DOI:** 10.3389/fnins.2016.00242

**Published:** 2016-05-31

**Authors:** Lindsay Tannenholz, René Hen, Mazen A. Kheirbek

**Affiliations:** ^1^Department of Pharmacology, Columbia UniversityNew York, NY, USA; ^2^Division of Integrative Neuroscience, New York State Psychiatric InstituteNew York, NY, USA; ^3^Department of Psychiatry, Columbia UniversityNew York, NY, USA; ^4^Department of Neuroscience, Columbia UniversityNew York, NY, USA; ^5^Department of Psychiatry, University of CaliforniaSan Francisco, CA, USA

**Keywords:** neurogenesis, dentate gyrus granule cells, GluN2B, synaptic plasticity, selective serotonin reuptake inhibitor (SSRI), antidepressants

## Abstract

Ablation of adult neurogenesis in mice has revealed that young adult-born granule cells (abGCs) are required for some of the behavioral responses to antidepressants (ADs), yet the mechanism by which abGCs contribute to AD action remains unknown. During their maturation process, these immature neurons exhibit unique properties that could underlie their ability to influence behavioral output. In particular, abGCs in the DG exhibit a period of heightened plasticity 4–6 weeks after birth that is mediated by GluN2B-expressing NMDA receptors. The functional contribution of this critical window to AD responsiveness is unclear. Here, we determined the behavioral and neurogenic responses to the AD fluoxetine (FLX) in mice lacking GluN2B-containing NMDA receptors in abGCs. We found that these mice exhibited an attenuated response to FLX in a neurogenesis-dependent behavioral assay of FLX action, while neurogenesis-independent behaviors were unaffected by GluN2B deletion. In addition, deletion of GluN2B attenuated FLX-induced increases in dendritic complexity of abGCs suggesting that the blunted behavioral efficacy of FLX may be caused by impaired differentiation of young abGCs.

## Introduction

Adult hippocampal neurogenesis is a process that results in the generation of new neurons in the dentate gyrus (DG) throughout life (Gross, 2000; Kempermann et al., 2015). After their birth, newborn cells progress through different developmental stages that are marked by unique gene expression patterns, morphological features, and electrophysiological properties (Zhao et al., 2008; Li et al., 2009; Drew et al., 2013, 2015; Piatti et al., 2013). Treatment with selective serotonin reuptake inhibitors (SSRIs) such as fluoxetine (FLX) can stimulate all stages of adult hippocampal neurogenesis. Increases in proliferation and survival have been observed leading to an overall increase in the number of adult-born granule cells (abGCs) integrating into the hippocampal circuit (Malberg et al., 2000; Santarelli et al., 2003; Encinas et al., 2006; Wang et al., 2008; David et al., 2009). Maturation of these abGCs is enhanced, as is a neurogenesis-dependent form of long-term potentiation (LTP) in the DG evoked by medial perforant path stimulation under intact GABAergic tone (ACSF-LTP; Wang et al., 2008). The timeline of neurogenesis effects match the drug's delayed onset of therapeutic action (Malberg et al., 2000; Santarelli et al., 2003; Wang et al., 2008). Importantly, when adult hippocampal neurogenesis is ablated, some of the behavioral effects of chronic FLX treatment are lost, revealing that abGCs not only respond to this treatment on a cellular level, but also contribute to the antidepressant (AD) action of the drug (Santarelli et al., 2003; Airan et al., 2007; Wang et al., 2008; David et al., 2009). While ablation methods have demonstrated the necessity of abGCs in certain AD-related behaviors, these techniques remove all abGCs from the circuit and do not allow for further dissection of chronic FLX's numerous effects on adult hippocampal neurogenesis. An alternative approach to studying this process would be to target and alter specific properties of young abGCs without removing these cells from the hippocampal circuit in order to determine which of these properties are critical for their contribution to the therapeutic action of FLX.

During the maturation process, abGCs exhibit distinct properties that could underlie their ability to influence behavioral output (Zhao et al., 2008; Li et al., 2009; Deng et al., 2010; Denny et al., 2012; Drew et al., 2013, 2015; Piatti et al., 2013; Danielson et al., 2016). In the period 4–6 weeks after birth, abGCs are more plastic than their mature counterparts—they exhibit a lower threshold for LTP induction and larger LTP amplitude (Ge et al., 2007). GluN2B antagonists block this enhanced plasticity, demonstrating that this unique electrophysiological property of abGCs is mediated by GluN2B-containing NMDA receptors (Snyder et al., 2001; Ge et al., 2007). This enhanced plasticity can be seen on the population level as well, underlying ACSF-LTP in the DG, which can be eliminated with ablation of neurogenesis or GluN2B antagonists (Snyder et al., 2001; Saxe et al., 2006). Conversely, increasing neurogenesis genetically or with chronic AD treatment increases levels of ACSF-LTP (Wang et al., 2008; Sahay et al., 2011a). Thus, a potential mechanism by which abGCs may contribute to AD behavioral efficacy is by increasing the number of highly plastic units in the DG circuit. Previously, we showed that deletion of GluN2B from abGCs in the DG abolished ACSF-LTP and impaired contextual fear discrimination, but had no impact on anxiety or AD-like behaviors (Kheirbek et al., 2012). Here, using the same approach, we tested whether GluN2B-containing NMDA receptors impact the AD action of FLX. We show that deletion of the GluN2B subunit significantly attenuates a neurogenesis-dependent behavioral response to FLX, and additionally may block FLX's ability to enhance young abGCs' maturation and subsequent integration into the hippocampal network.

## Materials and methods

### Mice

Experimental mice were homozygous for a loxP-flanked *Grin2b* allele (GluN2B^f/f^; von Engelhardt et al., 2008), homozygous for the ROSA26-STOP-floxed enhanced yellow fluorescent protein (EYFP) transgene (ROSA26^*fstopEYFP*/*fstopEYFP*^; Srinivas et al., 2001), and hemizygous for a NestinCreER^T2^ transgene (NCreER^T2^; Dranovsky et al., 2011). *Grin2b*, the gene coding for GluN2B, consists of 12 coding exons along with three non-coding exons located in the 5′-untranslated region (Klein et al., 1998). GluN2B^f/f^ mice have loxP sites surrounding exon 9 (the 6th coding exon) of *Grin2b*. In the presence of Cre, exon 9 gets excised. Western blotting performed using an antibody that targets the C-terminal domain downstream of exon 9 has shown decreased GluN2B expression following Cre recombination, demonstrating that excision of exon 9 results in the complete absence of GluN2B, not merely a truncated protein product (von Engelhardt et al., 2008). Ectopic expression has been reported in the NestinCreER^T2^ line and the impact this may have on our behavioral phenotype will be addressed in the discussion (Sun et al., 2014). Eight- to twelve-week-old mice were injected with tamoxifen (TMX, 3 mg dissolved in a solution of corn oil and 10% ethanol) or vehicle (corn oil/10% ethanol solution) intraperitoneally (IP) once per day for 5 consecutive days. Previous work with this NCreER^T2^ line did not reveal any significant sustained toxic effects of CreER^T2^ translocation to the nucleus in progenitor or daughter cells in the DG (Dranovsky et al., 2011; Kheirbek et al., 2012). Additionally, it has been shown that a brief pulse of TMX does not affect hippocampus-dependent behavior 6 weeks after administration of the drug (Sahay et al., 2011a). All experiments were approved and conducted in accordance to the guidelines of the Institutional Animal Care and Use Committee at Columbia University and the New York State Psychiatric Institute.

### Drugs

For behavior and neurogenesis measures, AD treatment began ~6 weeks after the last TMX injection. 5-bromo-2′-deoxyuridine (BrdU) was injected (150 mg/kg, IP dissolved in saline) once per day for 2 days just prior to the start of AD treatment. For dendritic morphology analysis, AD treatment began ~5 weeks after TMX administration and BrdU (75 mg/kg, IP dissolved in saline) was injected 4 times over 8 h the day before AD treatment began. For all mice, FLX (18 mg/kg/day in water) or vehicle (VEH, water) was delivered by oral gavage and in the drinking water (160 mg/mL) at a schedule of 5 consecutive days gavage followed by 2 consecutive days of water, then repeated for the duration of treatment.

### Behavioral testing

All behavioral experiments were conducted in male mice 18–24 weeks of age.

#### Novelty-suppressed feeding

In the novelty suppressed feeding (NSF) test, chronic AD treatment decreases latency to feed in the center of a novel arena after overnight food deprivation (Samuels and Hen, 2011). In mice with ablated neurogenesis, chronic AD treatment is no longer effective at lowering the latency to feed; therefore, NSF represents a neurogenesis-dependent behavioral assay of AD response (Santarelli et al., 2003; David et al., 2009). Mice were food restricted for 22–24 h and testing began when an animal was placed in the corner of a brightly lit (~1250 lux) plastic box (50 cm long × 28 cm wide × 15 cm deep) covered with the same type of bedding used in the animal's homecage. A single pellet of food (regular chow) had been positioned on a platform in the center of the box prior to the start of the test. The latency to eat (defined as the mouse sitting on its haunches and biting the pellet with the use of forepaws) was timed. Immediately after the latency was recorded, the food pellet was removed from the arena. Animals that did not eat within 10 min were censored during the statistical analysis. At the end of the session, animals were placed in their home cage and the amount of food consumed in 5 min was measured (home cage consumption). Each mouse was weighed before food deprivation and before testing to assess the percentage of body weight lost. Percent body weight lost and home cage consumption served as relative measures of animal hunger.

#### Tail suspension test

The tail suspension test (TST) is a behavioral despair test during which the mobility of mice suspended by their tail is measured. A depressed-like state is characterized by greater immobility while ADs are able to decrease immobility (Cryan et al., 2005). Sessions lasted 5 min and were video recorded. Scoring was done by an observer blind to induction and treatment.

#### Elevated plus maze

Mice were placed in the center of an elevated plus maze (EPM) consisting of both open and closed arms and were given 5 min to explore. The test was video recorded and activity was measured using TopScan software. Their preference for open (anxiogenic) or enclosed (anxiolytic) spaces was used to assess anxiety (Hogg, 1996).

### Immunohistochemistry

For all experiments, mice were perfused (4% paraformaldehyde), brains postfixed, cryoprotected, and sections (35 μm) of the entire DG were labeled for BrdU, glial fibrillary acidic protein (GFAP), neuronal nuclei (NeuN), doublecortin (DCX), Ki-67, or green fluorescent protein (GFP) [rat-anti-BrdU, 1:100 (Serotec); rabbit-anti-GFAP, 1:1500 (DAKO); mouse-anti-NeuN, 1:500 (Millipore); goat-anti-DCX, 1:500 (Santa Cruz); rabbit-anti-Ki67, 1:100 (Vector); chicken-anti-GFP, 1:500 (Abcam)], as previously described (Scobie et al., 2009). An experimenter blind to induction and treatment counted Ki67+, BrdU+, and DCX+ cells, as well as DCX+ cells exhibiting tertiary dendrites, in every sixth section throughout the DG. For colabeling of BrdU with NeuN or GFAP, confocal scans (FluoView1000; Olympus) at 40 × were taken of BrdU+ cells across the anteroposterior axis of the DG. For the morphological analysis of immature neurons, z-stack images of BrdU+/DCX+ cells were traced and imported into Adobe Illustrator CS5 where neurons were reconstructed using the tracing tool. Images of the reconstructed neurons were opened in Fiji (http://fiji.sc/Fiji) where dendritic length was measured using the freehand trace tool and Sholl analysis was conducted using the Sholl analysis plug-in with parameters previously described (Sahay et al., 2011a).

### Statistical analysis

Statistical significance was assessed by ANOVA or, in the case of planned comparisons, by unpaired two-tailed Student's *t*-test using Statview Software. Results in figures are mean ± SEM. Since many mice did not eat before the 10-min cutoff in the NSF test, information about their latency to feed was incomplete and these observations were censored. The Cox proportional hazards model was used to analyze this data so that we could both correctly account for the censored data points and evaluate the effect of multiple variables on survival (Hosmer et al., 2008). SAS Software was used to perform this analysis. A complete statistical summary for all experiments is included in Tables 1, 2.

**Table 1:** **Statistical analysis of the behavioral data**.

**Behavioral Test**	**N**	**Measurement**	**Statistical test**		**Factor**	**Statistic**	***p*-value**	**Figures**
Novelty suppressed feeding	15–18 mice/group	Hazard ratio	Cox proportional hazards model		Induction	$\chi_{\left(1\right)}^{2}$ = 1.4807	0.2237	Figure 2
					Treatment	$\chi_{\left(1\right)}^{2}$ = 21.7498	<**0.0001**	
					Interaction	$\chi_{\left(1\right)}^{2}$ = 5.3018	**0.0213**	
			Subgroup treatment effects	CTRL mice	Treatment	$\chi_{\left(1\right)}^{2}$ = 21.7498	<**0.0001**	Figure 2A
				iGluN2B^Nes^ mice	Treatment	$\chi_{\left(1\right)}^{2}$ = 5.1273	**0.0236**	Figure 2A
		Homecage consumption	2-way ANOVA		Induction	*F*_(1, 61)_ = 0.375	0.5424	Figure 2B
					Treatment	*F*_(1, 61)_ = 1.305	0.2578	
					Interaction	*F*_(1, 61)_ = 0.115	0.7357	
		% Weight change	2-way ANOVA		Induction	*F*_(1, 61)_ = 0.196	0.6596	Figure 2B
					Treatment	*F*_(1, 61)_ = 0.014	0.9047	
					Interaction	*F*_(1, 61)_ = 0.023	0.8811	
Elevated plus maze	15–18 mice/group	Open arm time	2-way ANOVA		Induction	*F*_(1, 61)_ = 0.098	0.7555	Figure 3A
					Treatment	*F*_(1, 61)_ = 0.643	0.4258	
					Interaction	*F*_(1, 61)_ = 0.031	0.8610	
		Open arm entries	2-way ANOVA		Induction	*F*_(1, 61)_ = 0.966	0.3295	Figure 3B
					Treatment	*F*_(1, 61)_ = 0.575	0.4511	
					Interaction	*F*_(1, 61)_ = 0.082	0.7756	
		Open arm distance	2-way ANOVA		Induction	*F*_(1, 61)_ = 0.358	0.5518	Figure 3C
					Treatment	*F*_(1, 61)_ = 0.214	0.6457	
					Interaction	*F*_(1, 61)_ < 0.0001	0.9989	
		Total Distance	2-way ANOVA		Induction	*F*_(1, 61)_ = 0.437	0.5112	Figure 3D
					Treatment	*F*_(1, 61)_ = 2.947	0.0911	
					Interaction	*F*_(1, 61)_ = 0.605	0.4398	
Tail Suspension Test	15–18 mice/group	Immobility	2-way repeated measures ANOVA		Induction	*F*_(1, 60)_ = 0.346	0.5585	Figure 3E
					Treatment	*F*_(1, 60)_ = 1.982	0.1644	
					Interaction	*F*_(1, 60)_ = 0.001	0.9706	
					Minute	*F*_(4, 240)_ = 114.851	<**0.0001**	
					Minute × induction	*F*_(4, 240)_ = 0.386	0.8187	
					Minute × treatment	*F*_(4, 240)_ = 4.136	**0.0029**	
					Minute × induction × treatment	*F*_(4, 240)_ = 0.124	0.9737	
		Immobility (last 2 min)	2-way ANOVA		Induction	*F*_(1, 60)_ = 0.023	0.8809	Figure 3F
					Treatment	*F*_(1, 60)_ = 10.402	**0.0020**	
					Interaction	*F*_(1, 60)_ = 0.027	0.8692	
			Planned Comparison *t*-test	CTRL mice	Treatment	*t*_(29)_ = 2.389	**0.0236**	
				iGluN2B^Nes^ mice	Treatment	*t*_(31)_ = 2.174	**0.0375**	

*p < 0.05 are highlighted in bold*.

**Table 2:** **Statistical analysis of neurogenesis measures**.

**Immunos**	**N**	**Measurement**	**Statistical test**		**Factor**	**Statistic**	***p*-value**	**Figures**
Ki-67	10–15 mice/group	Proliferation	2-way ANOVA		Induction	*F*_(1, 46)_ = 0.103	0.7492	Figure 4B
					Treatment	*F*_(1, 46)_ = 0.250	0.6198	
					Interaction	*F*_(1, 46)_ = 0.127	0.7231	
BrdU	14–16 mice/group	Survival	2-way ANOVA		Induction	*F*_(1, 54)_ = 7.897	**0.0069**	Figure 4C
					Treatment	*F*_(1, 54)_ = 25.980	<**0.0001**	
					Interaction	*F*_(1, 54)_ = 0.002	0.9615	
			Planned Comparison *t*-test	CTRL mice	Treatment	*t*_(26)_ = −4.472	**0.0001**	
				iGluN2B^Nes^ mice	Treatment	*t*_(28)_ = −3.143	**0.0039**	
BrdU/NeuN/GFAP	3 mice/group 19–39 cells/mouse	%BrdU+/NeuN+	2-way ANOVA		Induction	*F*_(1, 8)_ = 0.060	0.8133	Figure 4D
					Treatment	*F*_(1, 8)_ = 7.332	**0.0268**	
					Interaction	*F*_(1, 8)_ = 0.439	0.5261	
			Planned Comparison *t*-test	CTRLmice	Treatment	*t*_(4)_ = −0.870	0.4334	
				iGluN2B^Nes^ mice	Treatment	*t*_(4)_ = −2.770	0.0503	
		%BrdU+/GFAP+	2-way ANOVA		Induction	*F*_(1, 8)_ = 0.001	0.0775	
					Treatment	*F*_(1, 8)_ = 4.715	0.0617	
					Interaction	*F*_(1, 8)_ = 0.971	0.3534	
			Planned Comparison *t*-test	CTRLmice	Treatment	*t*_(4)_ = 1.226	0.2874	
				iGluN2B^Nes^ mice	Treatment	*t*_(4)_ = 2.292	0.0837	
DCX	8–16 mice/group	Immature Neuron Number	2-way ANOVA		Induction	*F*_(1, 41)_ = 0.125	0.7253	Figure 4F
					Treatment	*F*_(1, 41)_ = 6.851	**0.0124**	
					Interaction	*F*_(1, 41)_ = 0.578	0.4514	
			Planned Comparison *t*-test	CTRLmice	Treatment	*t*_(20)_ = −2.909	**0.0087**	
				iGluN2B^Nes^ mice	Treatment	*t*_(21)_ = −1.141	0.2667	
	6 mice/group	Tertiary Dendrites	2-way ANOVA		Induction	*F*_(1, 20)_ = 0.532	0.4742	Figure 4G
					Treatment	*F*_(1, 20)_ = 6.356	**0.0203**	
					Interaction	*F*_(1, 20)_ = 0.511	0.4832	
			Planned Comparison *t*-test	CTRLmice	Treatment	*t*_(10)_ = −2.022	0.0708	
				iGluN2B^Nes^ mice	Treatment	*t*_(10)_ = −1.506	0.1629	
BrdU/DCX	3 mice/group 2–5 cells/ mouse	Dendritic length	2-way ANOVA		Induction	*F*_(1, 34)_ = 1.724	0.1980	Figure 4H
					Treatment	*F*_(1, 34)_ = 2.222	0.1453	
					Interaction	*F*_(1, 34)_ = 0.995	0.3256	
			Planned Comparison *t*-test	CTRLmice	Treatment	*t*_(15)_ = −1.739	0.1025	
				iGluN2B^Nes^ mice	Treatment	*t*_(19)_ = −0.358	0.7246	
		Sholl Analysis	Repeated measures ANOVA—FLX treated mice		Induction	*F*_(1, 18)_ = 2.152	0.1596	Figure 4I
					Radius	*F*_(20, 360)_ = 24.446	<**0.0001**	
					Radius × Induction	*F*_(20, 360)_ = 1.174	0.2742	
			*t*-test	radius 70	Induction	*t*_(18)_ = 3.760	**0.0014**	
				radius 80	Induction	*t*_(18)_ = 2.202	**0.0410**	
			Repeated measures ANOVA—VEH treated mice		Induction	*F*_(1, 16)_ = 0.117	0.7363	Figure 4J
				Radius		*F*_(20, 320)_ = 16.139	<**0.0001**	
				Radius × Induction		*F*_(20, 320)_ = 0.187	>0.9999	

*p < 0.05 are highlighted in bold*.

## Results

**Figure 1:**
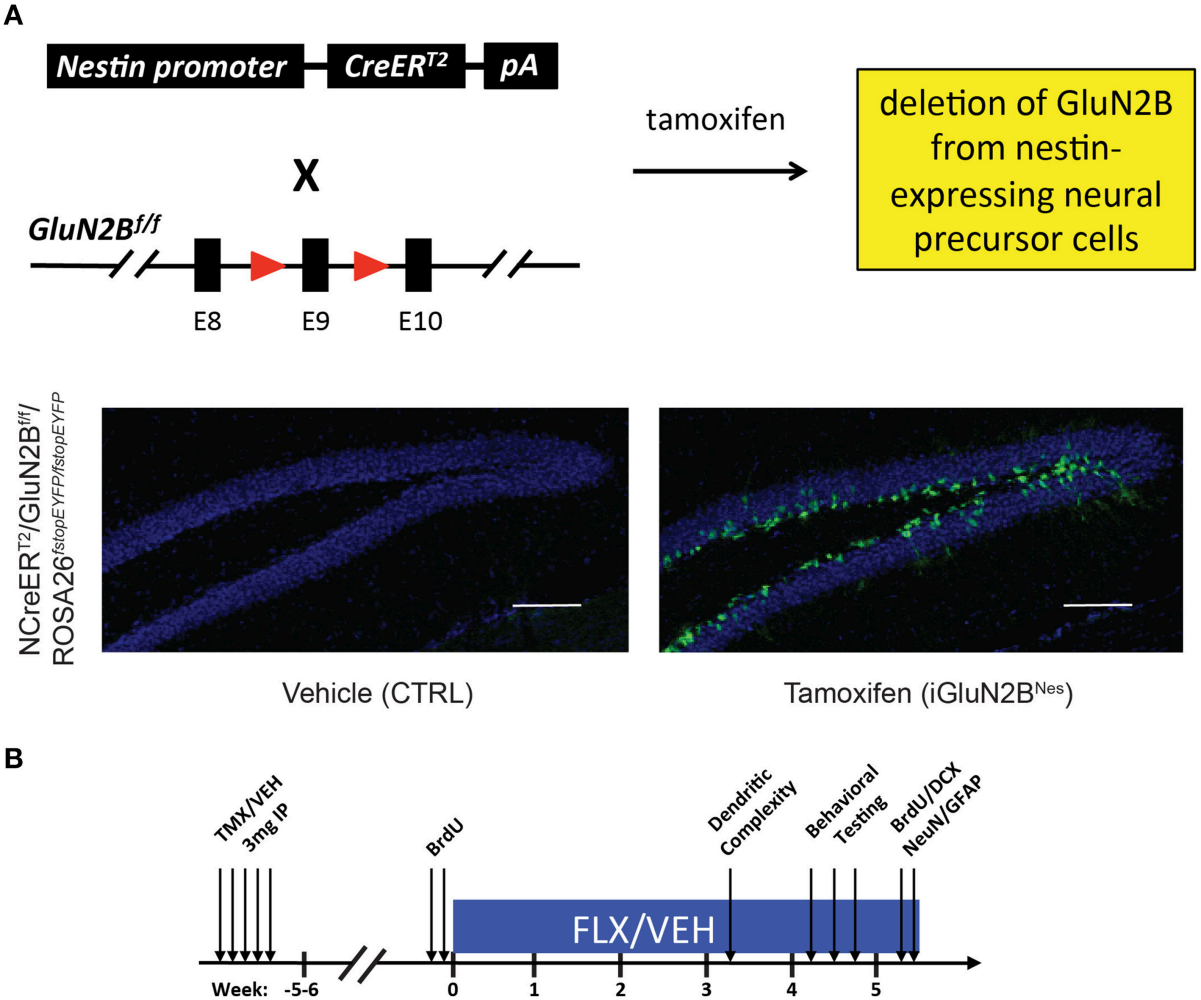
**Experimental Design. (A)** Genetic design for the deletion of GluN2B from abGCs. TMX administration into NCreER^T2^ × GluN2B^f/f^ × ROSA26^*fstopEYFP*/*fstopEYFP*^ mice eliminates the GluN2B subunit from NPCs and all of their progeny, while EYFP labels cells that underwent recombination thus acting as a surrogate for GluN2B deletion. Representative 10X images show cells are restricted to the lower portion of the granule cell layer near the subgranular zone, consistent with the expression pattern of immature abGCs (scale bar = 100 μm). **(B)** Experimental timeline: Adult NCreER^T2^ × GluN2B^f/f^ × ROSA26^*fstopEYFP*/*fstopEYFP*^ mice were injected with either TMX (iGluN2B^Nes^) or vehicle (CTRL). FLX or VEH treatment began 6 weeks later. Behavioral testing began after 4 weeks of chronic AD treatment. For dendritic complexity analysis, FLX or VEH treatment began 5 weeks after induction and mice were sacrificed after 3 weeks of AD treatment.

In order to examine the impact young abGCs' enhanced plasticity has on behavioral and neurogenic responses to chronic AD treatment, we specifically deleted the GluN2B subunit from those neurons. To both spatially and temporally control the deletion of GluN2B, we employed a double-transgenic mouse model in which mice express TMX-regulated Cre-recombinase under the control of the Nestin promoter (NCreER^T2^), while an encoding region immediately preceding the first transmembrane domain of GluN2B is flanked by loxP sites (GluN2B^f/f^, Figure 1A). To label cells that underwent recombination, mice were further bred to an inducible EYFP reporter line (ROSA26^*fstopEYFP*/*fstopEYFP*^). TMX injection in adult mice leads to deletion of GluN2B and expression of YFP in neural precursor cells (NPCs) and their subsequent progeny (iGluN2B^Nes^, Figure 1A). Littermates injected with vehicle served as our controls (CTRL, Figure 1A). We have previously characterized this mouse line and found that 6 weeks after TMX treatment, ~70% of immature, DCX+ granule cells in iGluN2B^Nes^ mice expressed EYFP and therefore were born from NPCs that have undergone recombination and presumably lack GluN2B (Kheirbek et al., 2012). This was confirmed electrophysiologically as slices from iGluN2B^Nes^ mice lack GluN2B-dependent ACSF-LTP (Kheirbek et al., 2012). Chronic FLX treatment began 6 weeks after TMX or vehicle administration in order to determine the effect of ADs in mice whose young abGC population lacks GluN2B (Figure 1B).

**Figure 2:**
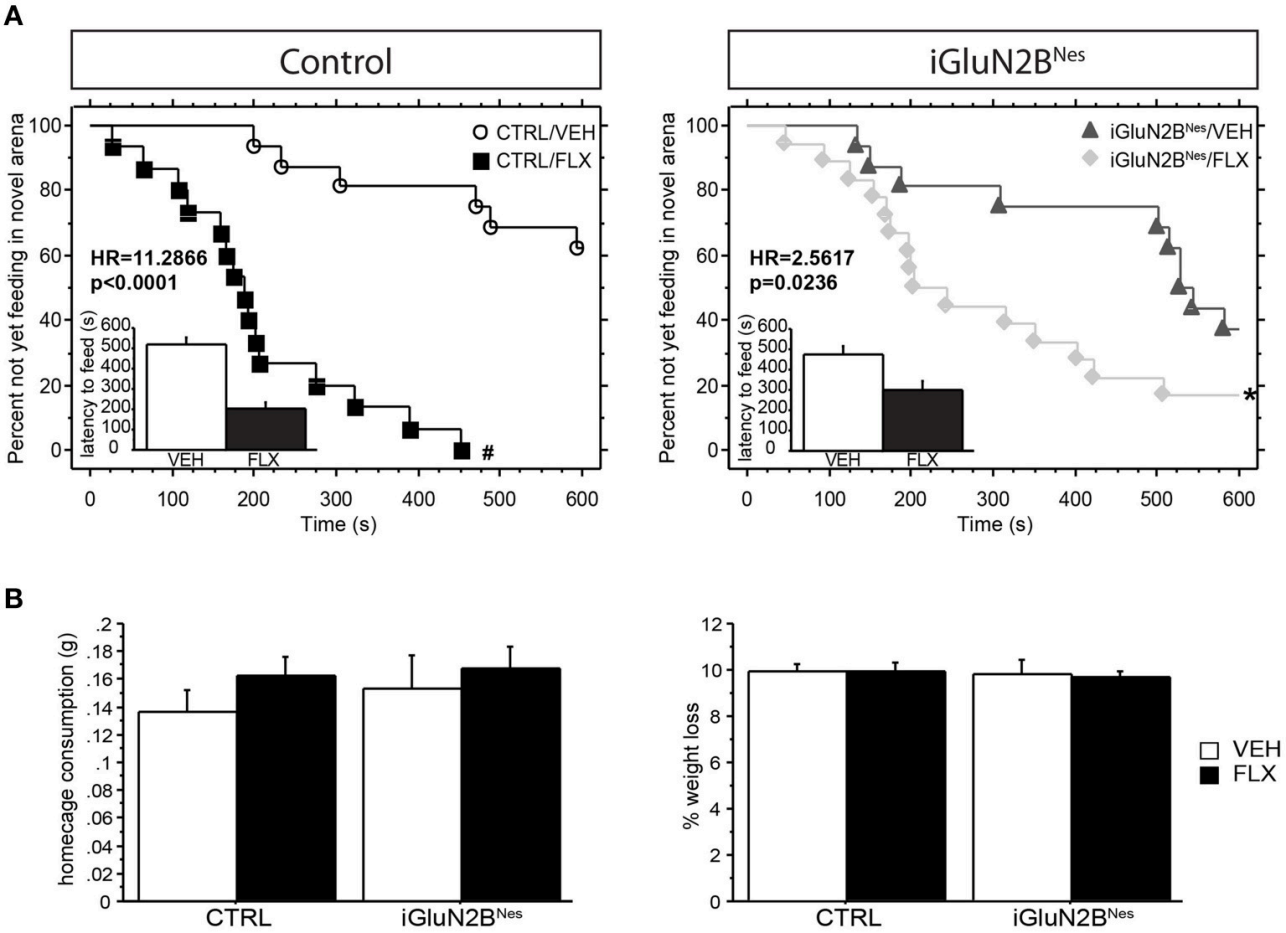
**Deletion of GluN2B attenuates the decrease in latency to feed in the NSF test after chronic FLX treatment. (A)** Cumulative survival plots comparing CTRL mice **(left)** or iGluN2B^Nes^ mice **(right)** treated with VEH or FLX. FLX significantly lowered the latency to feed in each group, with the greatest effect between treatment groups seen in CTRL mice. Insets show the average latency to feed in the novel arena, with those mice not feeding within the time limit of the test assigned a latency of 600 s. **(B)** No significant difference was observed in home cage consumption **(left)** or % weight change **(right)**. HR, Hazard Ratio. ^*^p < 0.05, ^#^*p* < 0.0001.

Four weeks after beginning chronic FLX treatment, iGluN2B^Nes^ mice were tested in anxiety and AD-like behavioral assays. Mice were tested in the NSF test, in which AD efficacy has been shown to require adult hippocampal neurogenesis (Santarelli et al., 2003; David et al., 2009). Analysis of latency to feed in this test revealed a significant interaction between induction and treatment leading us to look at comparisons across induction group (Table 1; see also Section Materials and Methods for a more detailed description of the statistical analysis used to analyze this data). FLX robustly lowered the latency to feed in the novel arena in CTRL mice with mice on FLX ~11 times more likely to eat at a given time than VEH mice (Figure 2A). For our iGluN2B^Nes^ group, FLX still significantly lowered the latency to feed in the novel arena, but the effect was more modest with mice receiving the drug only ~ 2.5 times more likely to eat at a given time than VEH mice (Figure 2B). For convenience we have also presented these data in a bar graph format (see insets in Figure 2A and corresponding legend). The observed differences in latency to feed were not due to changes in appetite as all groups exhibited similar levels of home cage food consumption and weight loss during the overnight food deprivation (Figure 2B). This indicates that loss of GluN2B from abGCs causes a blunting of the FLX effect in a neurogenesis-dependent behavioral assay of AD response.

**Figure 3:**
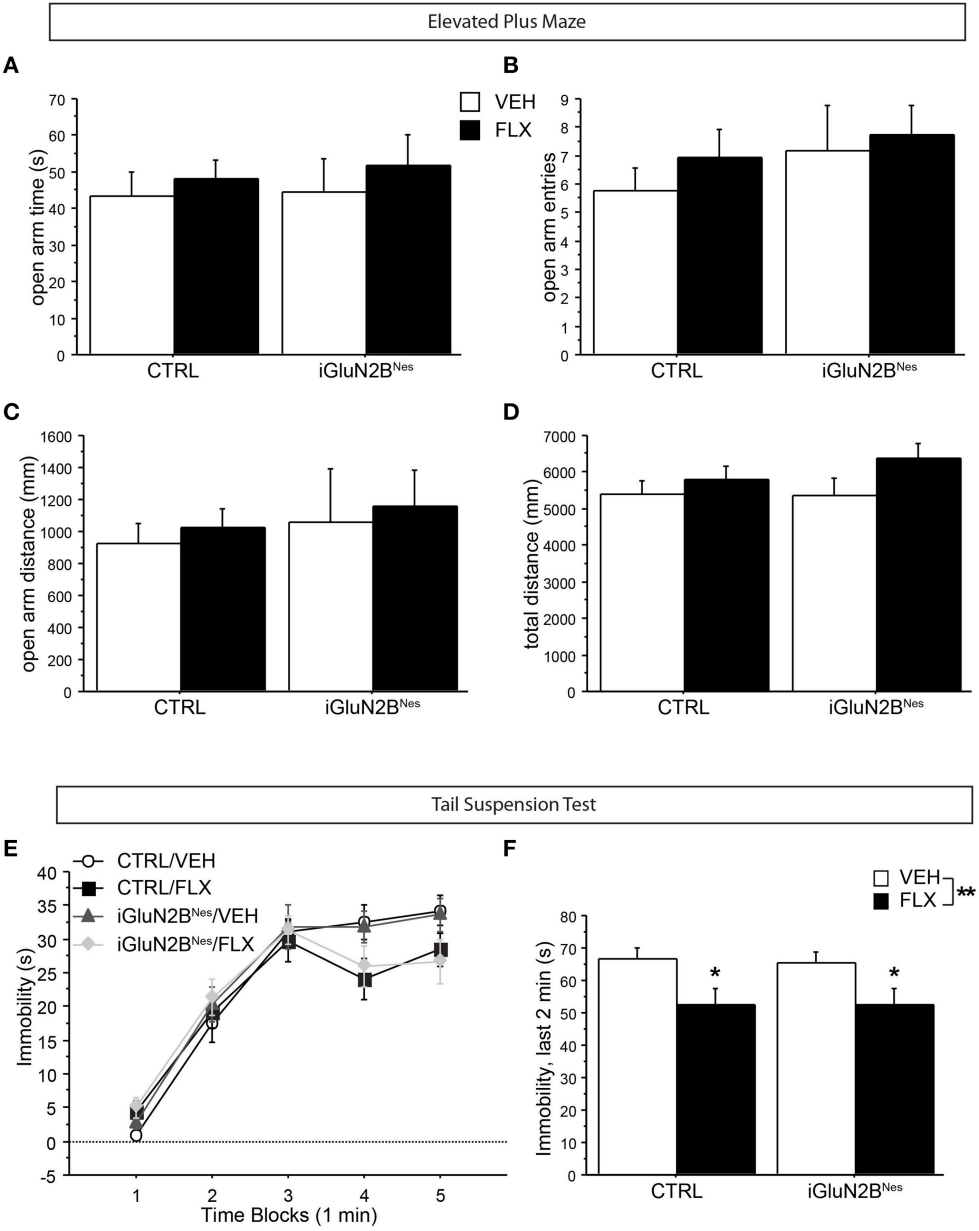
**GluN2B deletion did not impact anxiety-related behavior in the EPM, nor did it affect FLX's ability to lower immobility in the TST**. In the EPM, no difference among groups was observed in open arm exploration as measured by **(A)** time spent in the open arms, **(B)** number of open arm entries, or **(C)** distance traveled in the open arms. **(D)** The total distance traveled in the maze did not differ among groups. **(E)** In the TST, VEH-treated mice display increasingly higher levels of immobility as the test progresses, while FLX reduced immobility over time. **(F)** GluN2B deletion did not impact FLX's ability to lower immobility during the last 2 min of the test. ^*^p < 0.05, ^**^p < 0.01.

We also determined whether FLX treatment or GluN2B deletion altered anxiety-related behavior in the EPM. All groups showed similar levels of open arm exploration as measured by time spent in the open arms, open arm entries, and distance traveled in the open arms (Figures 3A–C). Furthermore, total exploration of the maze was similar for all the animals (Figure 3D). Together this shows that neither AD treatment nor GluN2B deletion altered anxiety-like behavior in this test.

Next, we tested the mice for FLX effects in the TST, a neurogenesis-independent behavioral assay of AD response (David et al., 2009). Mice on FLX were less immobile over time than VEH-treated mice (Figure 3E) and spent significantly less time immobile during the final 2 min of the test compared to VEH-treated mice with no significant interaction between induction and treatment (Figure 3F). This indicates that iGluN2B^Nes^ and CTRL mice respond similarly to FLX in a neurogenesis-independent assay of AD response.

**Figure 4:**
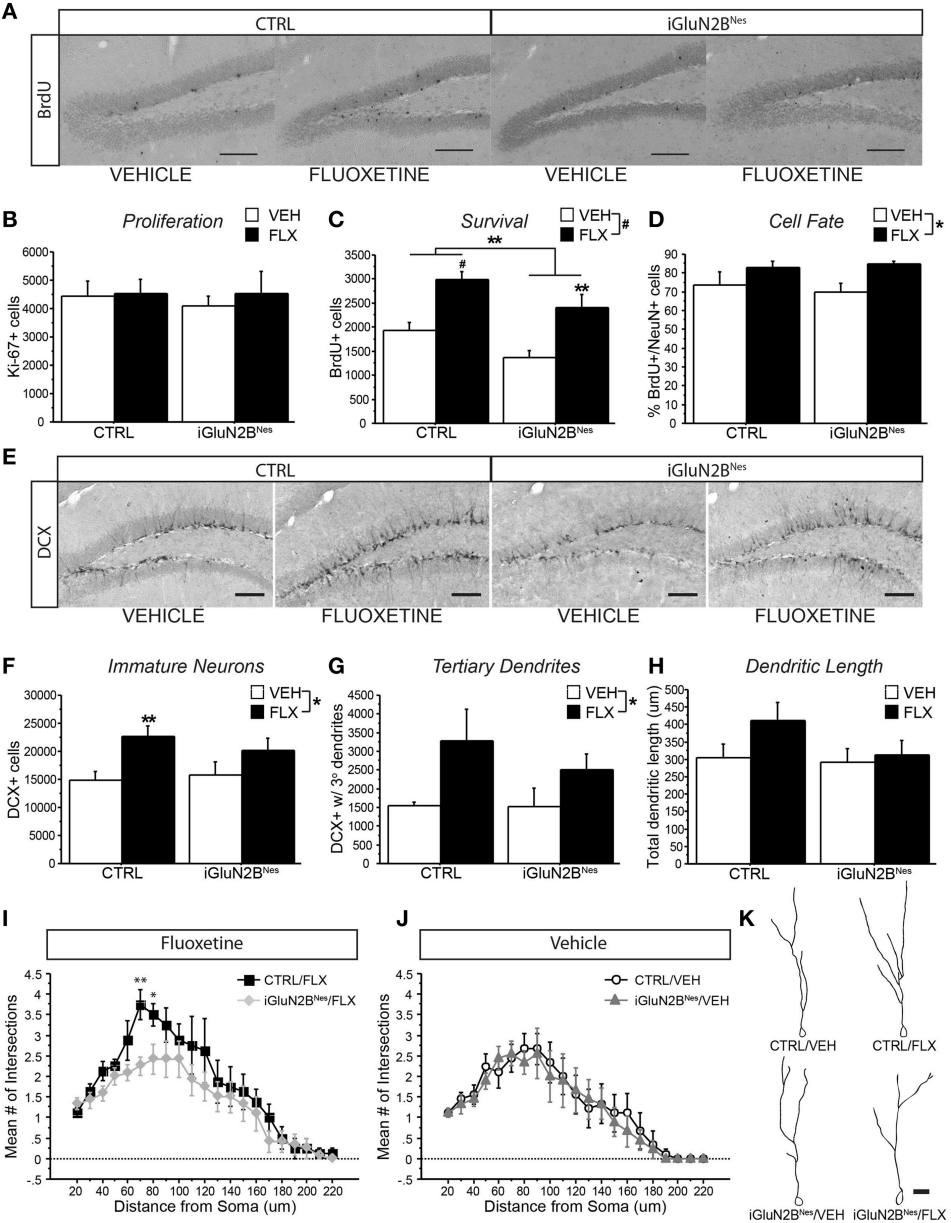
**FLX increased dendritic complexity in CTRL but not iGluN2B^**Nes**^ mice. (A)** Representative images of BrdU+ cells in the DG (scale bar = 100 μm) **(B)** No effect on proliferation was observed as measured by number of Ki-67+ cells. **(C)** FLX increased survival of abGCs in both CTRL and iGluN2B^Nes^ mice, though overall survival was lower in iGluN2B^Nes^ mice. **(D)** The majority of BrdU+ cells co-labeled with the neuronal marker, NeuN. FLX treatment increased the percentage of BrdU+ cells that were neuronal. **(E)** Representative images of DCX expression in the DG (scale bar = 100 μm). **(F)** The number of immature neurons and **(G)** immature neurons with tertiary dendrites increased following FLX treatment with the most robust effect seen in CTRL mice. **(H)** There was a trend for FLX to increase total dendritic length of 3-week-old DCX+ abGCs in CTRL mice, but not iGluN2B^Nes^ mice. **(I)** Sholl analysis revealed FLX increased dendritic complexity in CTRL mice, but not iGluN2B^Nes^ mice. **(J)** GluN2B deletion alone did not affect the dendritic complexity of 3-week-old DCX+ abGCs. **(K)** Representative tracings of 3-week-old abGCs (scale bar = 20 μm). ^*^p < 0.05, ^**^p < 0.01, #*p* < 0.0001.

We then assessed the neurogenic effects of FLX in iGluN2B^Nes^ and CTRL mice. The number of proliferating Ki-67+ cells did not significantly differ among groups, revealing that neither GluN2B deletion nor FLX treatment altered the number of new cells being produced at the time of sacrifice (Figure 4B). To assess the effect of FLX on survival, mice were injected with BrdU just prior to the start of AD treatment and surviving cells were counted 6 weeks later. Here, we found that the overall number of BrdU+ cells was lower after GluN2B deletion; however, FLX still increased survival in both CTRL and iGluN2B^Nes^ mice (Figures 4A,C). To determine the phenotype of BrdU+ cells, tissue sections were triple labeled for BrdU, NeuN (a neuronal marker), and GFAP (a glial marker). As expected, the majority of BrdU+ cells co-labeled with NeuN, though FLX treatment slightly increased the proportion of cells that were neuronal (Figure 4D). DCX, which is transiently expressed in newly generated neurons, was measured to quantify the number of immature neurons in the DG (Brown et al., 2003; Couillard-Despres et al., 2005). While FLX increased the number of DCX+ cells in CTRL mice, this effect was blunted in iGluN2B^Nes^ mice (Figures 4E,F). DCX+ cells undergo significant morphological changes as they mature. To determine if our manipulations altered this maturation process we counted the subset of DCX+ cells that had reached a more mature stage of development as exhibited by the presence of tertiary dendrites. FLX increased the number of DCX+ cells with tertiary dendrites in CTRL mice, but this effect was less pronounced in iGluN2B^Nes^ mice (Figure 4G). Finally, we measured the effect of FLX on dendritic structure by analyzing the dendrites of 3-week-old DCX+ cells. Once again, we found that FLX increased dendritic length and complexity in CTRL mice, but not in iGluN2B^Nes^ mice (Figures 4H–K).

**Figure 5:**
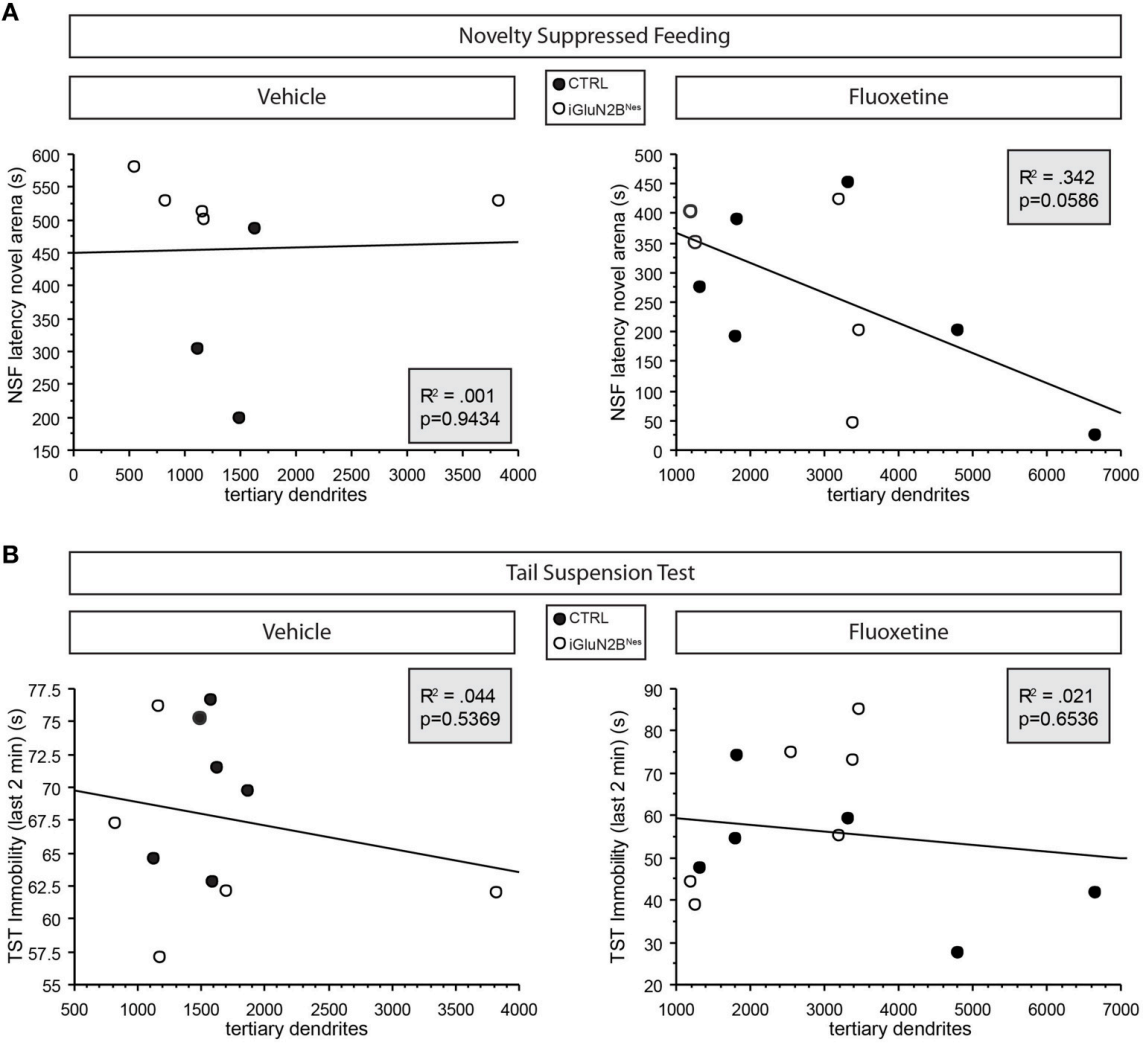
**Trend for dendritic complexity in FLX-treated mice to correlate with behavioral response in the neurogenesis-dependent NSF test. (A)** Higher numbers of immature neurons with tertiary dendrites tended to correlate with a lower latency to feed in NSF in FLX-treated mice **(right)** but not those mice that received VEH **(left)**. **(B)** Similar correlations were not evident in the neurogenesis-independent TST. Tertiary dendrite counts were obtained for 6 mice/group (see Table 2). For the NSF correlations, any mouse that had been censored was excluded from the correlations. For the TST correlations, 1 VEH-treated iGluN2B^Nes^ mouse was not included because he had to be removed from the TST for hanging onto his tail.

To determine if abGC maturation correlated with the behavioral response to FLX, we compared the number of DCX+ cells with tertiary dendrites in FLX-treated mice to outcomes in our FLX-responsive behavioral tests. We found a trend for greater tertiary dendrite number to correlate with a lower latency to feed in the NSF test (Figure 5A) in FLX-treated mice. When looking at the TST, which has been suggested to be independent of levels of neurogenesis (David et al., 2009), higher tertiary dendrite numbers were not predictive of a larger FLX response (Figure 5B). Tertiary dendrites were not related to behavioral outcomes in VEH-treated mice in either test (Figures 5A,B).

## Discussion

In this study, we have determined the consequence of altering young neurons' physiology on FLX efficacy without eliminating the young neurons themselves. By conditionally deleting the GluN2B subunit from abGCs of mice prior to the onset of AD administration, we revealed a blunting of the behavioral response to chronic FLX treatment. This suggests that eliminating abGCs' enhanced plasticity decreases their ability to influence DG output resulting in an AD response that is less robust than the response seen in CTRL mice. Control experiments revealed the specificity of this effect as GluN2B deletion did not impact the effect of FLX in a neurogenesis-independent behavioral assay of AD response (TST) or in an assay of anxiety that was insensitive to FLX in this strain of mice (EPM). While some studies in rodents do find that chronic AD treatment results in an anxiolytic phenotype in the EPM (Kurt et al., 2000; Bondi et al., 2008; David et al., 2009; Venzala et al., 2012; Samuels et al., 2015), this effect is not consistently seen (Durand et al., 1999; File et al., 1999; Griebel et al., 1999; Silva and Brandao, 2000; Borsini et al., 2002; Bondi et al., 2008; Oh et al., 2009; Venzala et al., 2012; Baek et al., 2015). This may be due to the manner in which different strains exhibit anxiety behavior in this test (Ducottet and Belzung, 2005) or the variability in testing conditions (Hogg, 1996).

The results presented here also revealed differences in the neurogenic effects of FLX in mice lacking GluN2B in abGCs. Interestingly, FLX effects on dendritic complexity were attenuated in iGluN2B^Nes^ mice, which also lack the full behavioral response to FLX. Our analysis indicated a trend for FLX-induced increases in dendritic complexity to be predictive of behavioral efficacy in the NSF test (Figure 5A). These results suggest that manipulating young neurons' plasticity attenuates FLX's ability to facilitate maturation thereby impacting the behavioral response. This supports the hypothesis that chronic FLX's efficacy may be dependent on accelerating the dendritic development of abGCs as they differentiate, which would serve to increase the number of young abGCs that are functionally integrated into the DG circuit (Wang et al., 2008).

The mechanism by which GluN2B deletion impairs the FLX-induced dendritic changes in young abGCs is not yet known, but one possibility is that it is through a brain-derived neurotrophic factor (BDNF)-dependent mechanism. Chronic SSRIs have been shown to increase BDNF in the DG (Nibuya et al., 1996; Samuels et al., 2015) and this effect has been shown to be reduced in mice that do not respond to SSRIs (Samuels et al., 2014, 2015). Mice with reduced BDNF signaling show impaired behavioral and neurogenic responses to AD treatment (Saarelainen et al., 2003; Monteggia et al., 2004; Sairanen et al., 2005). In addition, mice with abGCs lacking the BDNF receptor, tropomyosin receptor kinase B (TrkB), show reduced dendritic maturation, impaired ACSF-LTP, and lack behavioral and neurogenic responses to chronic AD-treatment (Bergami et al., 2008; Donovan et al., 2008; Li et al., 2008). These effects on survival, dendritic complexity, LTP, and AD sensitivity are quite similar to the ones observed in iGluN2B^Nes^ mice at baseline or following chronic FLX treatment suggesting a link between our manipulation of GluN2B expression and BDNF signaling. BDNF signaling can upregulate the mRNA and protein expression of NMDA receptor subunits, increase trafficking of NMDA receptors to the cell membrane, and enhance the activity of NMDA receptors via phosphorylation (Lin et al., 1998; Slack et al., 2004; Caldeira et al., 2007). Through this enhancement of NMDA receptor activity, BDNF may potentiate activity-dependent development, while the elimination of GluN2B-containing NMDA receptors would likely limit the extent to which BDNF could regulate this process. An alternative possibility is that NMDA receptor signaling can positively modulate the BDNF signaling cascade (Zafra et al., 1991; Springer et al., 1994), which could then act in an autocrine manner to activate TrkB receptors on abGCs and impact dendritic complexity.

Our analysis of neurogenesis revealed two other surprising results. First, we found that chronic FLX treatment did not increase proliferation in either CTRL or iGluN2B^Nes^ mice. This is in contrast to the many studies that have observed a link between chronic AD treatment and increased proliferation (reviewed in Hanson et al., 2011). This is likely due to the mixed background of the mice used in the present study, which includes the C57BL/6 strain. C57 mice have high levels of proliferation at baseline (Kempermann et al., 1997a) and do not display an increase in proliferation in response to FLX in the absence of prior stress (David et al., 2009).

Second, we found that the survival of abGCs in iGluN2B^Nes^ mice was lower than in CTRL mice, which was not seen in our previous study (Kheirbek et al., 2012). The present study differed in that the mice used here were subject to oral gavage 5 days of every week, which leads to prolonged increases in corticosterone levels (Dalm et al., 2008). One possibility is that the mice in this study may have only become sensitive to the influence GluN2B deletion has on survival with the addition of this chronic stressor. Despite the overall lower survival seen in iGluN2B^Nes^ mice, FLX was still effective at increasing survival relative to VEH-treated animals. It is interesting that the stress from oral gavage was sufficient to reveal the action of FLX on survival, but not proliferation. However, survival and proliferation are regulated by distinct mechanisms, and as such it is possible to affect one and not the other. For example, environmental enrichment and hippocampus-dependent learning tasks increase cell survival without effecting proliferation (Kempermann et al., 1997b; Gould et al., 1999). Also, an increase in survival with no effect on proliferation was found in C57BL/6 mice that had received chronic FLX treatment via daily IP injections and were thus exposed to a comparable amount of stress as our mice (Couillard-Despres et al., 2009). Examination of the fate of surviving BrdU+ cells showed that the percentage of BrdU+ cells that co-labeled with NeuN increased with FLX treatment, mimicking effects on cell fate seen after enrichment (Dranovsky et al., 2011).

It should be noted that the SGZ is not the only neurogenic region in the adult mouse brain (Zhao et al., 2008). Using this particular genetic approach means that GluN2B will also be deleted form newborn neurons in the subventricular zone (Grubb et al., 2008). However, ablation of adult-born olfactory interneurons achieved using a NCreER^T2^ mouse line similar to the one used here did not significantly affect olfaction (Imayoshi et al., 2008). Innate olfactory responses and odor discrimination were intact as was the mouse's ability to acquire and retain odor-associated memory. Thus, it seems unlikely that the behavioral paradigms used in this study would be affected by the deletion of GluN2B from adult-born olfactory interneurons.

An additional caveat is that recombination also occurs ectopically outside of the neurogenic areas with our NCreER^T2^ mouse line (Sun et al., 2014). The most prominent ectopic recombination occurred in the cerebellum (where GluN2B mRNA is not expressed in adult mice; Monyer et al., 1994). The other areas with significant recombination were the CA1 and CA3 regions of hippocampus, thus some effects of GluN2B deletion may be due to loss of the subunit in these areas. However, our behavioral results in the NSF, EPM, and TST tests, phenocopies mice with targeted ablation of hippocampal neurogenesis, further linking the behavioral phenotype we observe to our manipulation of GluN2B in abGCs (Wu and Hen, 2014). In the future, retroviral approaches or local delivery of TMX will provide more targeted methods for localizing behavioral effects to abGCs.

It is particularly interesting that eliminating young neurons' enhanced plasticity does not completely block the FLX actions that are considered to be neurogenesis-dependent. This may be due to the fact that iGluN2B^Nes^ mice do not entirely lack FLX-induced increases in levels of neurogenesis, only the ability to add highly plastic units to the DG circuit. Alternatively, the remaining effects of ADs may be independent of neurogenesis. FLX has shown some residual behavioral effects following ablation similar to the attenuation in FLX efficacy we see in our iGluN2B^Nes^ mice (Wu and Hen, 2014). There is also accumulating evidence that mature granule cells in the DG are involved in the effects of ADs too (Samuels et al., 2015).

Possibly linking these two hypotheses, young abGCs have been suggested to modulate the activity of mature GCs within the DG. In the absence of neurogenesis, activity in the DG increases (Burghardt et al., 2012; Lacefield et al., 2012; Ikrar et al., 2013), whereas increasing levels of neurogenesis or stimulating young abGCs decreases DG activity (Ikrar et al., 2013; Drew et al., 2015). While the exact mechanism for this is unknown, it has been hypothesized that young abGCs may differentially modulate the local DG circuits (Sahay et al., 2011b; Lacefield et al., 2012). Our immunohistochemical analysis indicates that abGCs in iGluN2B^Nes^ mice still survive and mature into neurons, and thus may still be capable of influencing the hippocampal network in a manner that does not require enhanced plasticity. Future experiments will be required to determine whether these cells lacking GluN2B can impact local and downstream circuits like control cells.

While we observe an effect of GluN2B deletion in our study, it is possible that the same manipulation would not impact behavior in middle-aged or aged mice given that they display lower levels of neurogenesis than the young-adult mice used here (Hamilton et al., 2013). This consideration highlights the impact different experimental parameters can have on the study of neurogenesis. Differences in age, as well as strain, species, and ablation or enhancement techniques used likely underlie the conflicting reports in the literature in relation to adult hippocampal neurogenesis' function. For example, some studies show that reducing neurogenesis enhances anxiety (Revest et al., 2009; Snyder et al., 2011), while others find the opposite result with lower levels of neurogenesis associated with less anxiety (Fuss et al., 2010; Groves et al., 2013). Our behavioral results are in line with findings indicating that neurogenesis does not impact anxiety or antidepressant-like behavior at baseline, but does contribute to the behavioral response to antidepressants (Santarelli et al., 2003; Airan et al., 2007; Sahay and Hen, 2007; Surget et al., 2008; David et al., 2009; Sahay et al., 2011a).

Considering neurogenesis' impact on mood raises the question of how changes within the DG can influence downstream circuitry relevant for stress and anxiety. Recent findings suggest the hippocampus may be functionally segregated along the dorsoventral axis as a result of regional variation in anatomical connectivity (Fanselow and Dong, 2010; Kheirbek et al., 2013; Tannenholz et al., 2014). Targeting ablation of neurogenesis to the dorsal or ventral DG revealed that dorsal abGCs are required for learning a contextual fear discrimination task, whereas ventral abGCs were necessary for the anxiolytic/AD effects of FLX in the NSF test (Wu and Hen, 2014). While our manipulation targeted abGCs along the entire axis of the DG, it would be interesting to further dissect the role of this unique form of plasticity by specifically targeting ablation of GluN2B to either the dorsal or ventral DG.

Our results reveal that adult neurogenesis, and in particular the unique electrophysiological properties of young abGCs, contributes to the efficacy of AD treatment. These experiments, along with future studies aimed at further understanding the ways in which abGCs participate in ADs' mechanism of action, may help uncover novel avenues for therapeutic interventions.

## Author contributions

LT, RH, and MK designed the experiments and wrote the paper. LT performed the experiments and analyzed the data.

## Funding

LT was supported by the National Institute of Mental Health (F31 MH100842). RH is supported by the National Institute of Mental Health (R37 MH068542 [MERIT]), the National Institute on Aging (R01 AG043688), the National Institute of Neurological Disorders and Stroke (R01NS081203), NYSTEM (C029157), and the Hope for Depression Research Foundation (RGA 11-024). MK was supported by a NIH grant (K01MH099371), a NARSAD Young Investigator Award from the Brain & Behavior Research Foundation, and NYSTEM (C029157).

### Conflict of interest statement

RH receives compensation as a consultant for Roche and Lundbeck. The other authors declare that the research was conducted in the absence of any commercial or financial relationships that could be construed as a potential conflict of interest.
